# Diffuse Axonal Injury in the Rat Brain: Axonal Injury and Oligodendrocyte Activity Following Rotational Injury

**DOI:** 10.3390/brainsci10040229

**Published:** 2020-04-10

**Authors:** Michela Losurdo, Johan Davidsson, Mattias K. Sköld

**Affiliations:** 1 Department of Neuroscience, Karolinska Institute, 171 77 Stockholm, Sweden; mattias.skold@ki.se; 2 Department of Molecular Medicine, Università degli Studi di Pavia, 27100 Pavia, Italy; 3 Department of Mechanics and Maritime Sciences, Chalmers University of Technology, 412 96 Gothenburg, Sweden; johan.davidsson@chalmers.se; 4 Department of Neuroscience, Section of Neurosurgery, Uppsala University, 751 85 Uppsala, Sweden

**Keywords:** diffuse axonal injury, traumatic brain injury, myelin degradation, oligodendrocyte progenitor cell, olig2

## Abstract

Traumatic brain injury (TBI) commonly results in primary diffuse axonal injury (DAI) and associated secondary injuries that evolve through a cascade of pathological mechanisms. We aim at assessing how myelin and oligodendrocytes react to head angular-acceleration-induced TBI in a previously described model. This model induces axonal injuries visible by amyloid precursor protein (APP) expression, predominantly in the corpus callosum and its borders. Brain tissue from a total of 27 adult rats was collected at 24 h, 72 h and 7 d post-injury. Coronal sections were prepared for immunohistochemistry and RNAscope^®^ to investigate DAI and myelin changes (APP, MBP, Rip), oligodendrocyte lineage cell loss (Olig2), oligodendrocyte progenitor cells (OPCs) (NG2, PDGFRa) and neuronal stress (HSP70, ATF3). Oligodendrocytes and OPCs numbers (expressed as percentage of positive cells out of total number of cells) were measured in areas with high APP expression. Results showed non-statistically significant trends with a decrease in oligodendrocyte lineage cells and an increase in OPCs. Levels of myelination were mostly unaltered, although Rip expression differed significantly between sham and injured animals in the frontal brain. Neuronal stress markers were induced at the dorsal cortex and habenular nuclei. We conclude that rotational injury induces DAI and neuronal stress in specific areas. We noticed indications of oligodendrocyte death and regeneration without statistically significant changes at the timepoints measured, despite indications of axonal injuries and neuronal stress. This might suggest that oligodendrocytes are robust enough to withstand this kind of trauma, knowledge important for the understanding of thresholds for cell injury and post-traumatic recovery potential.

## 1. Introduction

Traumatic brain injury (TBI) is an important public health problem and is the leading cause of disability and death among young adults [1,2]. A thorough understanding of the pathological mechanisms behind TBI, such as myelin and oligodendrocyte loss and regeneration, and the time window in which such mechanisms might occur, sets the basis for developing timely therapeutic approaches that might aid in recovering tissue damage and in restoring function.

When the head is exposed to rotational accelerations, common in sports-related accidents and in road traffic accidents, the brain lags behind in a non-homogeneous way [3,4], producing shearing and strain of the brain tissues. Shearing and strain is especially seen at the borders between tissues with different stiffness properties, e.g., the border between the corpus callosum and cortex, where large concentrations of injured cells are found [5]. 

Rotational accelerations of the head, here referred to as rotational TBI, may lead to tissue loadings that could produce primary axotomy. [6,7] While this type of axotomy might occur during severe trauma, it is far more common that axotomy evolves over time after the trauma (secondary axotomy), with the axons undergoing Wallerian degeneration [7,8,9]. At the time of injury, axons may be stretched and undergo cytoskeletal damage due to neurofilament compaction and mechanoporation of the cell membrane, which results in membrane leakage, ionic disturbances, and osmotic imbalance [5,10]. Cytoskeletal damage results in impaired axoplasmic transport and proteins such APP and NF accumulate, leading to the formation of axonal bulbs [11]. APP thus serves as a useful marker for investigating axonal injury [12,13]. In the days to weeks following injury, such progressive disorganization of the axonal cytoskeleton and protein accumulation leads to secondary axotomy [5,7,11]. Other than axonal injury, these events are thought to be related to oligodendrocyte apoptosis, neuronal somata perturbations and death [14,15]. Heat shock proteins such as HSP70 and Activating Transcription Factor 3 (ATF3) are induced following stress of different nature (e.g., trauma, ischemia, hyperthermia and hypoxia) [16,17] and have been found to be upregulated early following TBI in humans and in animals, hence forming useful markers to detect areas of cell stress following trauma [14,18,19]. 

Axonal injury is thought to cause myelin damage by direct structural damage to the adjacent myelin membrane when the axon undergoes primary axotomy, often initiating at the nodes of the Ranvier and para-nodal region [20]. Alternatively, myelin can be damaged indirectly following the release of glutamate and other excitatory amino acids from neurons that have been mechanically disrupted following trauma, resulting in calpain-mediated Myelin Basic Protein (MBP) degradation. MBP is a major structural protein of the myelin sheath and its degradation—which is seen as early as 48 h—might lead to myelin sheath instability and demyelination [21,22,23]. Moreover, MBP mRNA expression was shown to be decreased following shear stress in the context of the direct mechanical stimulation of Schwann cells [24].

Myelin degradation is not only a consequence of axonal injury; the demyelination of non-injured axons might occur following TBI, when TBI induces a loss of oligodendrocytes [20,25], since oligodendrocytes function in producing and maintaining the myelin sheaths. Oligodendrocytes are lost as they undergo apoptosis, which is triggered by excitotoxicity, oxidative stress, and caspase-3-mediated cell death mechanisms. These events have been widely described occurring following TBI [26,27,28], resulting from glutamate release from internal stores in the neuron cell body, calcium overload and mechanoporation. The loss of oligodendrocytes leads to myelin sheath collapse and degradation, eventually leading to axon demyelination. Demyelinated axons are prone to damage, so the loss of oligodendrocytes might also lead to secondary axonal injury [29,30,31,32].

As oligodendrocyte loss following TBI takes place, immunostaining with markers specific for oligodendrocytes, such as anti-Olig2, might reveal a decreased number of such cells [33,34]. Olig2 is a basic helix–loop–helix transcription factor restricted to the oligodendrocyte lineage. It is expressed throughout the life of the oligodendrocyte, from its early development to maturity [35], hence anti- Olig2 is a good marker to evaluate the oligodendrocyte population.

Oligodendrocyte death and demyelination are known to be major triggers for oligodendrocyte progenitor cells’ (OPCs) activation and proliferation [36]. OPCs are adult resident progenitor cells present throughout the adult brain with the potential to proliferate and differentiate given the right stimuli. Activated OPCs slowly migrate to the site of injury, where they differentiate into mature myelin-producing oligodendrocytes. For this to occur, OPCs must switch from resting to active state, which shows enlarged cell bodies and hypertrophied processes. Then, OPCs will attempt to re- myelinate axons and heal the damage [37,38]. Re-myelination is defined as the process by which damaged myelin sheath is replaced by newly formed myelin [38,39,40,41]. Moreover, other than from OPCs, oligodendrocytes develop from stem cells in the subventricular zone [42,43].

Thus, if in the first moments following injury it might be possible to observe a decreased number of Olig2+ cells secondary to mature oligodendrocyte death, it is likely that then—as the process of OPCs’ activation and proliferation takes place—overall Olig2+ cells might increase in number [33,34]. To differentiate Olig2+ oligodendrocytes from Olig2+ OPCs, markers specific to OPCs can be used, such as anti-NG2. The NG2 is a chondroitin sulfate proteoglycan present on the plasma membrane of OPCs. NG2+ cells have been shown to increase in number following spinal cord injury and TBI [44,45]. 

Given the role that OPC proliferation has in re-myelination, and the clinical importance of myelination in terms of signal conduction and axonal integrity, additional knowledge of the rate and magnitude of myelin degeneration, oligodendrocyte loss and oligodendrocyte reactive proliferation, may contribute to the improved diagnosis and treatment of TBI. From this perspective, we aim at assessing how the myelin and cells involved in its formation and degradation—oligodendrocytes—react to TBI induced by angular acceleration of the head of rats. 

## 2. Materials and Methods

### 2.1. Animal Preparation

The experiments were carried out on 27 male Sprague-Dawley rats (weighing 440.8 ± 102.3 g), divided into exposed groups and sham and exposed to different survival times (24 h, 72 h, 7 d). Of the 27 rats, 18 (weighing 425.2 g ± 45.3 g) were included in both the quantitative and qualitative analysis, equally divided among sham and exposed groups (Table 1). All the animals were deeply anesthetized by 2.4 mL/kg intra-abdominal injections of a mixture of Fentanyl, Midazolam and Domitor. Thereafter, the subjects were given 0.2 mL/kg intra-muscular injections every 30 min until pre-surgery, trauma and post-surgery were carried out.

All the work performed was in accordance with the Swedish National Guidelines for Animal Experiments and approved by the Stockholm Animal Care and Use Ethics Committee (Stockholm, Norra Djurfösöksetiska Nämnd). Ethical permit numbers: NG22/10, N248/11, N81/13.

### 2.2. Experimental Setup

**Figure 1:**
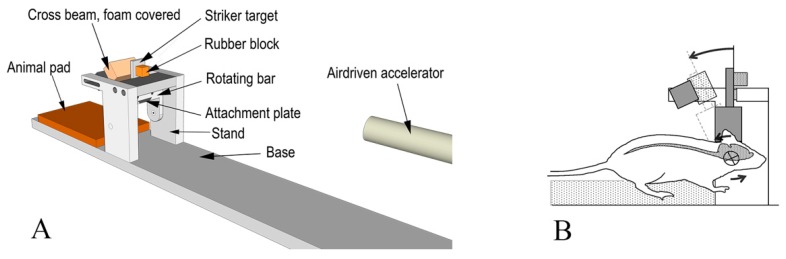
Experiment setup scheme. (**A**) Oblique view of the test device. (**B**) Side view of the test subject placed within the device. The central nervous system (CNS) is schematically depicted, and the center of rotation highlighted. The arrows indicate the direction of movement.

The rotational TBI was produced using a model described by Davidsson and Risling [46]. This model allows for sagittal plane rotational acceleration of the head of rats (Figure 1) and simulates forehead to hard structure impacts. The model allows to assess graded levels of inertia-induced brain injuries without major contusion.

In brief, a midline incision was made through the skin and periosteum on the skull vault to expose the bone. A skull cap was glued to the bone and an attachment plate was fastened by means of two screws to the skull cap. Then, the attachment plate was inserted and secured to a rotating bar that can rotate freely around its horizontal axis. The resulting center of rotation was located 1 mm below the head base and 5 mm anterior to the front of the foramen magnum. Both sham and trauma-exposed groups underwent these procedures. 

During trauma, a striker was accelerated in a specially designed air-driven accelerator and was made to hit a rubber block attached to a striker target attached to the rotating bar. The impulse produced subjected the rotating bar and the animal head to a short sagittal plane rearward rotational acceleration. The rotational acceleration magnitude was selected by modifying the striker speed, which was varied by means of modifying air pressure in the accelerator. The acceleration selected in this study ranged between 0.96–2.2 Mrad/s^2^ while the durations of the accelerations were identical (0.4 ms). Such an angular acceleration range was selected based on results from previous investigations on the same model of injury, which show a linear increase in APP expression from values of 1 Mrad/s^2^ [46]. After trauma, the attachment plate was removed, the skin was made to cover the skull cap, and eight to ten sutures closed the incision. 

The animals were kept under standard animal care conditions for survival times of 24 h (*n* = 6 for traumatized and *n* = 4 for sham), 72 h (*n* = 7 for traumatized and *n* = 4 for sham) and 7 d (*n* = 3 for traumatized and *n* = 3 for sham).

### 2.3. Dissection and Immunohistochemistry

The brains were removed and split into several units and fresh frozen on dry ice. In this study, a minimum of nine coronal cryostat sections were cut from a unit at 1.5–0.5 mm relative to bregma (referred to as frontal sections), from a unit at −3.0 to −6.0 mm relative to the bregma (referred to as occipital sections). The sections were put onto slides and allowed to airdry for 30–60 min at room temperature (20 °C). The slides where fixed in 4% buffered formalin for 10 min and subsequently rinsed in 0.01 M PBS twice for 10 min. The sections were incubated overnight in a humid chamber at 4 °C with one or more primary antibodies. All primary antibodies (Table 2) were diluted in 0.01 M PBS containing 5% donkey serum, 5% bovine serum albumin, 0.3% triton. The sections were then soaked in 0.01 M PBS twice for 10 min and incubated for 40 min at room temperature with the secondary antibodies (Cy3 donkey anti-mouse; Dy488 donkey anti-rabbit; Cy2 donkey anti-goat). All secondary antibodies were diluted in 0.01 M PBS containing 0.3% triton. All slides were incubated for five minutes with DAPI stain (1 μL DAPI stock solution in 4 mL 0.01 M PBS) to visualize cell nuclei and served as a background anatomical map, and were then soaked in 0.01 M PBS twice for 10 min. Finally, the slides were mounted in Mowiol 4-88 (Polyscience) and cover slipped. 

Primary antibodies were used or combined in the following ways: anti-APP antibody was used to detect axonal injuries, anti-MBP antibody and anti-Rip antibody were used to detect myelin changes, anti-Olig2 antibody was used to detect the presence of oligodendrocyte lineage cells. The anti-MBP antibody was combined either with anti-Olig2 or anti-APP antibody to assess the myelination status in conjunction with oligodendrocyte losses or with the degree of axonal injury. An anti-NG2 antibody, a chondroitin sulphate antibody, was used to detect OPCs, and it was combined with anti-Occludin-1 antibody to differentiate OPCs from blood vessels. An anti-A2B5 antibody was used to detect immature glial precursor cells, and thus to detect early differentiating OPCs. Anti-HSP70 and anti-ATf3 were used to detect neuronal somata stress, in combination with anti-APP. 

### 2.4. RNAscope^®^ ISH

In situ hybridization was performed on fresh frozen sections, as directed by the manufacturer (Advanced Cell Diagnostics, Hayward, CA, USA) [47]. Custom RNAscope^®^ target-specific oligonucleotide (ZZ) probes were designed by Advanced Cell Diagnostics (Advanced Cell Diagnostics, Newark, CA, USA) targeting nucleotides 375-1986 (NM_001100557.1) for Olig2 and 1516-2531 (NM_012802.1) for PDGFRa of Rattus Norvegicus (Table 3). Slides were counterstained with cresyl violet, mounted in Mowiol 4-88 (Polyscience) and cover slipped. The rn-PPIB gene targeting nucleotides 95-830 (NM_022536.2) was used as positive control probe.

### 2.5. Microscopic and Statistical Analyses

**Figure 2:**
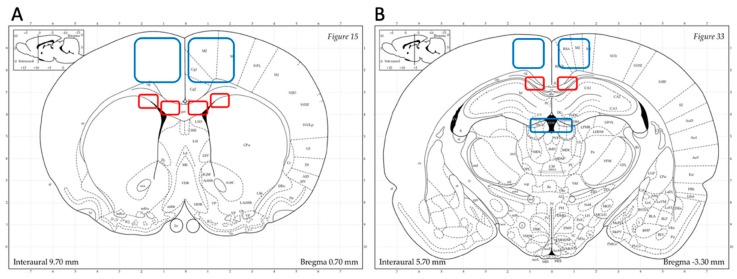
Regions of interest. Highlighted areas represent the systematically analyzed regions throughout the work. (**A**) Frontal brain sections focus on corpus callosum (in this study referred to as CC “subventricular zone” (SVZ) and CC “border”), dorsal cortex. (**B**) Occipital brain sections focus on corpus callosum (in this study referred to as CC “occipital”), dorsal cortex and habenular nuclei. (Atlas source: Paxinos, George and Charles Watson. The rat brain in stereotaxic coordinates: hard cover edition. Access Online via Elsevier, 2006. Online tool by Matt Gadica).

Immunohistochemistry (IHC) sections were examined using a Nikon Ni-Eclipse microscope with Andora Zyla fluorescence camera and Nikon DS-Fi2 light camera, and Nikon Eclipse E600 confocal microscope. Regions of interest were defined based on evidence of injuries from previous TBI studies using the rotational model adopted in this study. For frontal sections, analysis focused on the corpus callosum (just dorsal of the lateral ventricles—in this study referred to as CC “SVZ”—and ventral of the cingulum and dorsal of the interface with the caudoputamen—in this study referred as CC “border”) and dorsal cortex (Figure 2A). For occipital sections, analysis focused on the corpus callosum, dorsal cortex and habenular nuclei (Figure 2B). 

A thorough histological examination and photographic documentation was done in order to perform a qualitative analysis of the adopted rotational brain injury model. During microscopy, the observer localized the region of interest and described at 10× magnification the gross pattern of each antibody expression, i.e., whether it was expressed or not, and if its expression clustered in specific areas. Two to three overview photographs per side—depending on the extension of the ROI—were taken at 10× magnification. At 20× magnification, descriptions about each antibody’s expression density and signal intensity were made. Photographs were taken on each side for each ROI, and those showing the most consistent anatomical orientation among the different subjects and slides were chosen for analysis. 60× magnification was used to observe single NG2+ OPC morphology. All photographs were taken with same camera settings (objective, magnification, exposure times).

Quantitative analysis of Rip and APP expression was performed on IHC slides. As for Rip, the images taken at 20× with the same camera settings, for each ROI bilaterally, were analyzed in terms of mean grey value (MGV) by means of Fiji ImageJ (Wayne Rasband, NIH http://imagej.nih.gov/ij). APP expression was analyzed by manually counting the APP+ axon terminals in each ROI bilaterally. 

RNAscope^®^ slides were examined using Nikon Ni-Eclipse microscope with light camera. All images used to quantify Olig2+ and PDGFRa+ cell numbers were taken at 20× magnification with uniform camera settings. For these images, a quantitative analysis was performed using the software Fiji ImageJ (Wayne Rasband, NIH http://imagej.nih.gov/ij) following the guidelines suggested by Advance Cell Diagnostics for slides processed with RNAscope^®^ [48]. The parameters used for interpreting the results and statistical analysis were the percentage of positive cells in the region of interest (ROI). 

The observer had experience in microanatomy, was aware of the regions to be documented and the antibody of use, but was blinded to the treatment and time point being analyzed. 

For each of the quantitative analyses, a two-tail unpaired *t*-test (GraphPad Prism software) was performed to establish differences between the sham and exposed groups, while one-way ANOVA test was used for establishing differences between the exposed groups at different survival times. Statistical significance was assumed for *p* values ≤ 0.05. All the results are presented as average values ± standard deviation. 

## 3. Results

### 3.1. Axonal Injury: Anti-APP Antibody

**Figure 3:**
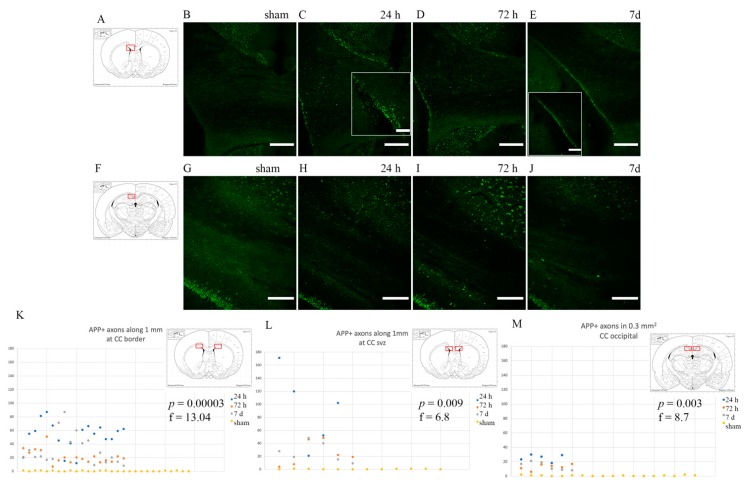
APP expression in the corpus callosum. Fluorescence microscopy photographs of the corpus callosum (coronal sections) at around 1mm from bregma (top row) and around −3 mm from bregma (bottom row). (**A**,**F**) The highlighted area refers to the area represented in the photographs on the same row. (**C**–**E**) Following injury, axonal bulbs localize mostly at the borders between the corpus callosum and grey matter. Insert show details of the CC-lateral ventricle border area. (**H**–**J**,**M**) Moving occipitally, APP expression is comparatively less. (**B**,**G**) Sham animals do not show APP accumulation in the axonal terminals at the regions of interest. (**K**–**M**) Scatter plots showing the amount of APP+ profiles in each ROI (y-axis represents the number of APP+ profiles; x-axis represents each observation). APP expression is maximal at 24 h and decreases thereafter, with *p* ≤ 0.05 at one-way ANOVA analysis. Scale bar = 300 µm (top row); scale bar = 500 µm (bottom row); scale bar = 150 µm (C insert); scale bar = 500 µm (E insert).

Intense APP staining was observed in rotational TBI-exposed subjects as opposed to shams, which did not express any APP, frontally in the corpus callosum close to the borders with both the cortex, caudoputamen and the lateral ventricles (Figure 3). However, in the occipital portion of the corpus callosum (around −3 mm from Bregma), APP-positive bulbs appeared to be more scarcely distributed (*p* ≤ 0.05) than in the frontal corpus callosum (about 1.3 mm from Bregma) (Figure 3K–M). Otherwise, throughout the corpus callosum, APP-positive profiles appeared scattered and smaller in size than at its borders. APP+ profiles appeared more abundant at 24 h and 72 h than at 7 d (*p* ≤ 0.05) (Figure 3K–M). 

### 3.2. Oligodendrocyte Lineage Cells: Anti-Olig2 Antibody and Olig2 mRNA

**Figure 4:**
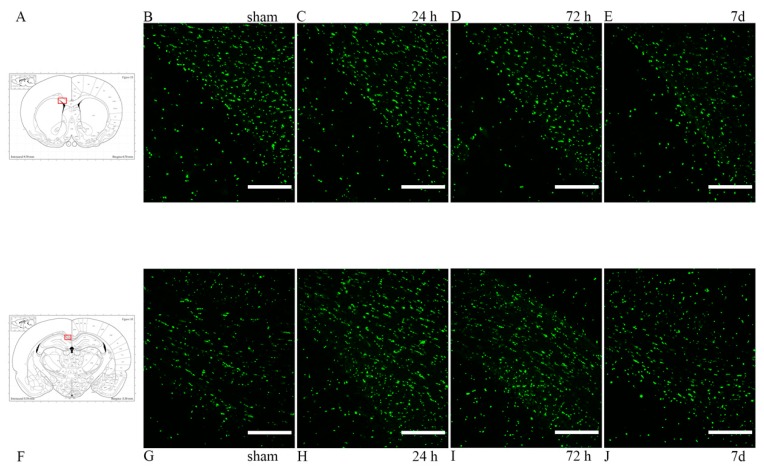
Olig2 expression in the corpus callosum. (**A**,**F**) The highlighted area refers to the area represented in the photographs on the same row. (**B**–**E**) Olig2 expression in the corpus callosum, at its interface with the lateral ventricle at around 1 mm from bregma. (**G**–**J**) Olig2 expression in the corpus callosum at around −3 mm from bregma. Scale bar = 200 µm.

**Figure 5:**
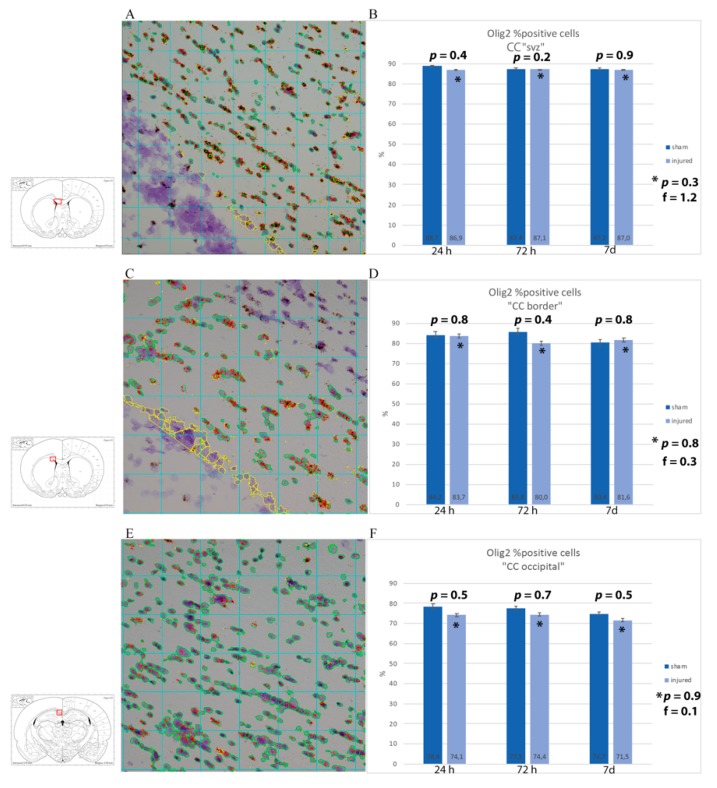
RNAscope analysis with Olig2 mRNA probe of corpus callosum. (**A**,**C**,**E**) Sample of RNAscope light microscopy picture of injured subjects set for analysis with Fiji ImageJ. The insert shows the region of interest squared in red, namely (**A**) CC just dorsal of the subventricular zone (CC “SVZ”), (**C**) area of the corpus callosum at around 1 mm from Bregma at the border with the caudoputamen, and (**E**) corpus callosum at around −3 mm from Bregma. All the cells considered for the analysis are circled in green, while in red are the Olig2 probe clusters. In yellow is what has been manually excluded because it was considered as background/not part of the region of interest. (**B**,**D**,**F**) Results from the image analysis are presented as averages of the percentages of the number of cells expressing Olig2 mRNA out of the total number of cells from each subject in the ROI. While a trend with a lower number of Olig2 was observed in injured as compared to sham, such a difference was not statistically significant at any timepoint. * *p* indicates results from one-way ANOVA analysis, while *p* is from Student *t*-test.

Oligodendrocyte population was studied through anti-Olig2 antibody. In the corpus callosum, both in the frontal and in the occipital sections, Olig2 was expressed at baseline levels, with no clear differences seen between sham and injured animals by visual inspection (Figure 4). In order to have a detailed understanding of the changes in oligodendrocyte lineage cell densities following injury, the percentage of cells expressing Olig2 mRNA was estimated in regions of interest (corpus callosum dorsal of the lateral ventricles, at its border with the caudoputamen and occipitally just lateral of the midline) from light microscopy pictures of RNAscope^®^ slides. By visual inspection, a difference in amount of mRNA being expressed could be observed, with more abundant and larger mRNA clusters in sham animals at longer survival times. These changes were not statistically significant, although the analysis provided that there appears to be a trend of higher percentage of Olig2 mRNA-expressing cells in sham as compared to injured animals and a decrease in the percentage of positive cells in injured rats at 7 d as compared to 24 h when considering only the smaller ROI at the border of the corpus callosum and caudoputamen. (Figure 5).

### 3.3. Oligodendrocyte Progenitor Cells: Anti-NG2, Anti-A2B5 Antibodies and PDGFRa mRNA

**Figure 6:**
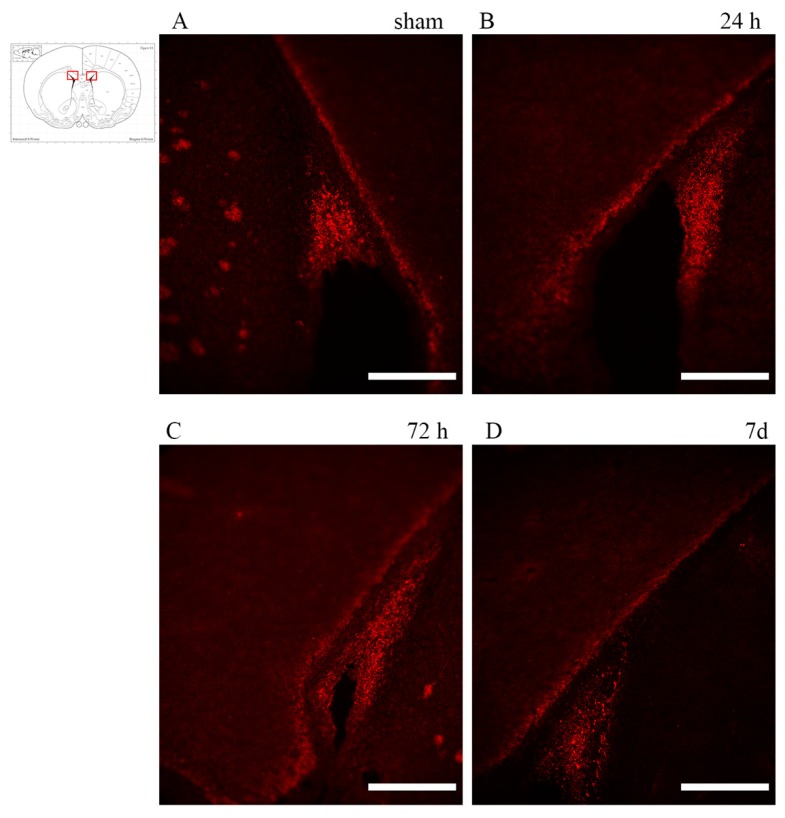
A2B5 expression in the subventricular zone. (insert) The highlighted area refers to the regions of interest represented in the microscopy pictures. (**A**–**D**) Abundant A2B5 positivity can be seen in the subventricular zone, but is apparently decreased at 7d. Scale bar = 200 µm.

OPCs were labelled using anti-NG2 antibody and immature glial precursor cells (so to detect early differentiating OPCs) using anti-A2B5 antibody. Analysis of the brain sections of rotational TBI subjects revealed that A2B5+ cells localized predominantly at the subventricular zone, apparently decreasing at 7 d as compared to 24 h (Figure 6).

**Figure 7:**
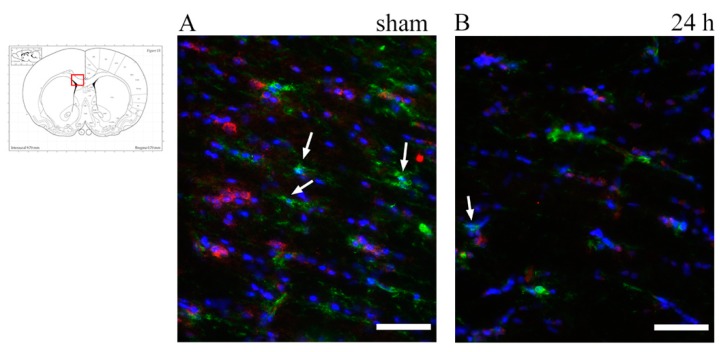

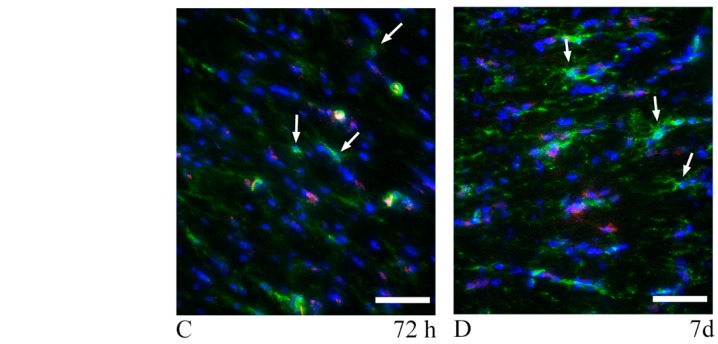
NG2 expression in the corpus callosum at around 1 mm from bregma. (**A**–**D**) Fluorescence microscopy photographs show NG2+ oligodendrocyte progenitor cells in the corpus callosum. Double staining with Occludin (red) was done to distinguish NG2+ (green) vessel walls from oligodendrocyte progenitor cells (OPCs). DAPI+ nuclei are stained in blue. Arrows show NG2+/DAPI+/Occludin− OPCs, which appear scattered throughout the corpus callosum, with no apparent difference between sham and trauma-exposed subjects. Insert: the highlighted area refers to the area represented in (**A**–**D**). Scale bar = 50 µm.

**Figure 8:**
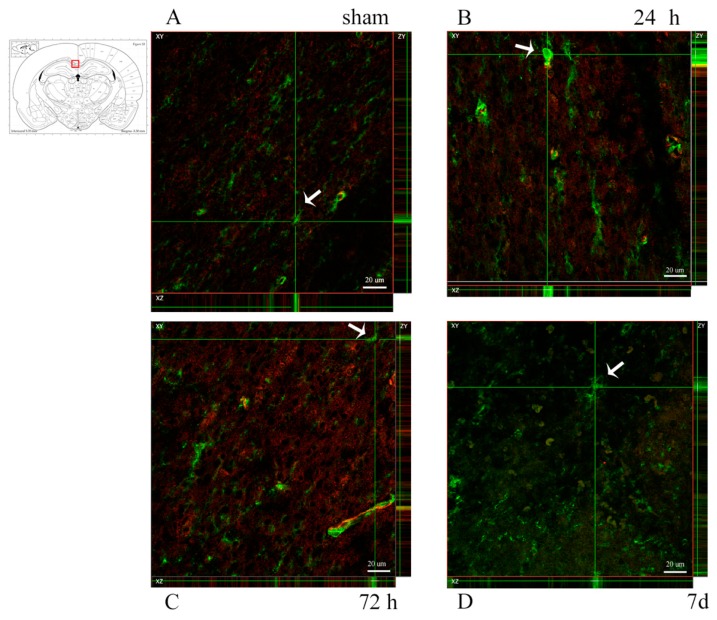
NG2 expression in the corpus callosum at around -3 mm from bregma. (**A**–**D**) Confocal microscopy photographs show NG2+ oligodendrocyte progenitor cells in the corpus callosum. Double staining with Occludin-1 (red) was done to distinguish NG2+ (green) vessel walls from OPCs. Arrows indicate OPCs as shown by the expression of NG2 on the XYZ planes. NG2+ OPCs are scattered throughout the corpus callosum, with no apparent difference between sham and trauma-exposed subjects. Insert: the highlighted area refers to the area represented in (**A**–**D**). Scale bar = 20 µm.

**Figure 9:**
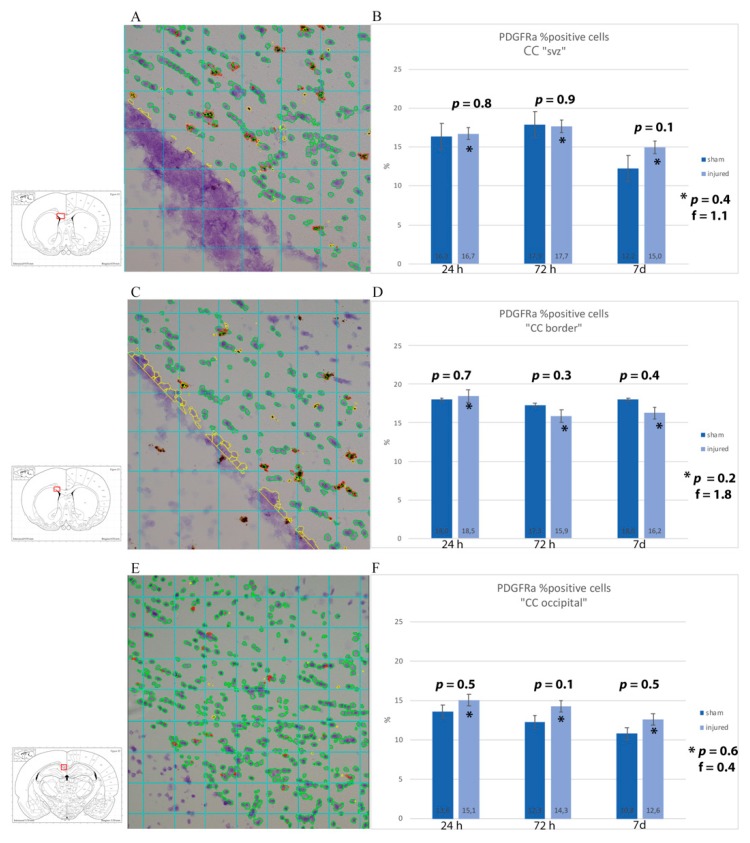
RNAscope analysis with PDGFRa mRNA probe of corpus callosum. (**A**,**C**,**E**) Sample of RNAscope light microscopy picture of injured subjects set for analysis with Fiji ImageJ. The insert shows the region of interest squared in red, namely (**A**) CC just dorsal of the subventricular zone (CC “SVZ”), (**C**) area of the corpus callosum at around 1 mm from Bregma at the border with the caudoputamen, and (**E**) corpus callosum at around −3 mm from Bregma. All the cells considered for the analysis are circled in green, while in red are the PDGFRa probe clusters. In yellow is what has been manually excluded because it was considered as background/not part of the region of interest. (**B**,**D**,**F**) Results from the image analysis are presented as averages of the percentages of the number of cells expressing PDGFRa mRNA out of the total number of cells from each subject in the ROI. While a trend of an increased number of PDGFRa-expressing cells was observed in injured as compared to sham when analyzing the whole width of the corpus callosum at around 1 and −3 mm from bregma, this is not true when considering a smaller ROI of the corpus callosum at around 1 mm from bregma. Moreover, such differences were at no timepoint statistically significant. * *p* indicates results from one-way ANOVA analysis, while *p* is from Student *t*-test.

To identify OPCs, slides were co-labelled with anti-NG2 antibody and anti-Occludin antibody (specific for the vessel’s tight junctions) so as to distinguish NG2+ OPCs from NG2+ vessel walls. Visual analysis was performed at fluorescence and confocal microscopes. Scattered NG2+ OPCs were found in the corpus callosum regions of interest. The difference between injured and sham animals could not be readily identified (Figure 7 and Figure 8). For the quantitative analysis of OPCs, percentage of cells expressing PDGFRa mRNA (out of all of the cells counterstained by cresyl violet) were counted at all timepoints and regions of interest (Figure 9). Results were compared by means of two-tail unpaired *t*-test between sham and injured animals, and by means of one-way ANOVA test between injured animals at different timepoints. Statistical analysis revealed decreased percentage of cells expressing PDGFRa mRNA both by 7 d (7 d < 24 h) and in the injured animals as compared to shams in the frontal brain sections. In more occipital sections (around −3 mm from bregma), instead, higher percentages of PDGFRa mRNA-expressing cells were recorded in injured rats than in shams. However, none of these trends showed statistical significance. 

### 3.4. Myelin Pathology: Anti-MBP and Anti-Rip Antibodies

Myelin changes following rotational TBI were investigated by means of anti-MBP antibody, which recognizes the 21.5 kDa MBP isoform, and the anti-Rip antibody, which recognizes the 2,3 cyclic nucleotide 3-phosphodiesterase in mature, myelinating oligodendrocytes and myelin sheaths. 

**Figure 10:**
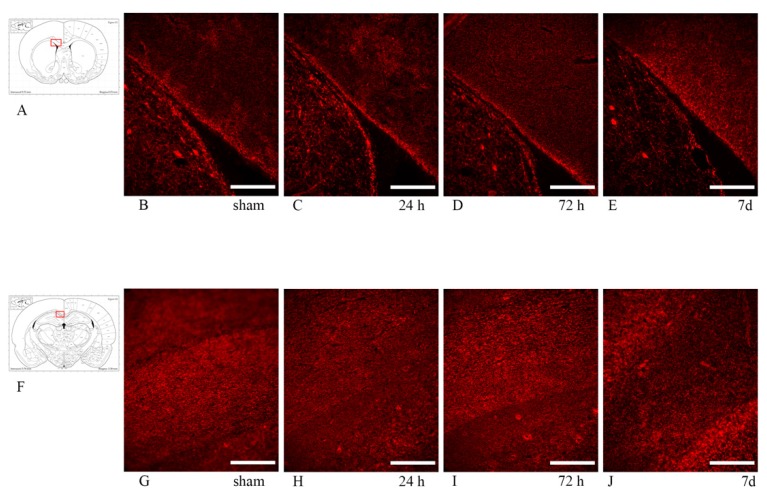
Rip (top row) and MBP (bottom row) expression in the corpus callosum. Fluorescence microscopy photographs of the corpus callosum (coronal sections) in different regions of interest. MBP and Rip stainings identify the myelin sheath. (**A**,**F**) The highlighted area refers to the area represented in each row. (**B**–**E**) anti-Rip antibody expression in the corpus callosum at around 1 mm from bregma, adjacent to the lateral ventricle. Rip expression appears similar between sham and exposed animals at all timepoints. (**G**–**J**) anti-MBP antibody expression in the corpus callosum at around −3 mm from bregma. No clear difference is noted between sham and exposed animals at all timepoints. Scale bar = 200 µm.

In the corpus callosum, both MBP and Rip expression appeared inhomogeneous, but similar between sham and injured animals at all timepoints, so that no clear difference between sham and injured rats was observed (Figure 10). 

**Figure 11:**
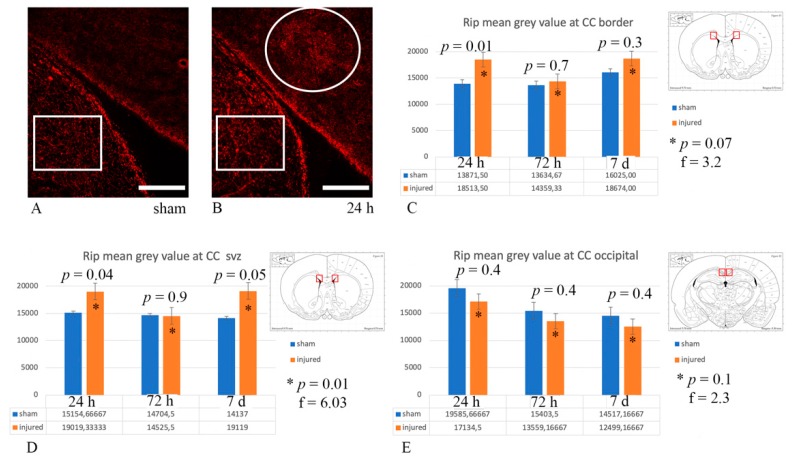
Rip expression analysis. (**A**,**B**) Examples of Rip expression in the CC “SVZ” in the sham and 24 h injured rat, respectively. The circle highlights an area of Rip expression, which is perceived as brighter, reflecting higher mean grey values (16,000 in (**B**)) at the quantitative analysis. Similarly, the rectangles highlight the area of the caudoputamen used as control for mean grey value, which is higher (15,600 and 17,200 in (**A**) and (**B**), respectively) than in the sham CC (13,400 in (**A**)). Scale bar = 200 µm. (**C**–**E**) The graphs represent quantitative analysis of Rip expression expressed as mean grey values. The ROI for each graph is represented by the red highlighted area in the schematic inserts. (**C**) Statistically significant (*p* ≤ 0.05) difference of Rip expression was found between sham and injured animals at 24 h, as well as across injured animals, at the CC border with the caudoputamen. (**D**) Statistically significant (*p* ≤ 0.05) difference in Rip expression was also found between sham and injured animals at 24 h and 7 d, as well as across injured animals, at the CC “SVZ”. * *p* indicates results from one-way ANOVA analysis, while *p* from Student *t*-test.

However, when performing quantitative analysis of Rip expression as mean grey value of the microscopy picture from each ROI bilaterally, this revealed changes in Rip expression (*p* ≤ 0.05) between sham and exposed animals at 24 h, both at the border of the CC with the caudoputamen and at the CC “SVZ”, as well as at 7 d at the CC “SVZ” (Figure 11).

### 3.5. Neuronal Stress: Anti-HSP70 and Anti-ATF3 Antibodies

**Figure 12:**
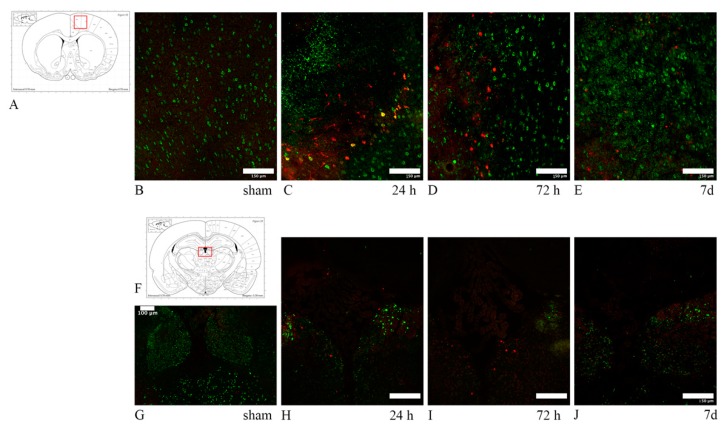
HSP70/ATF3/APP expression reflecting neuronal stress. (**A**,**F**) The highlighted area refers to the area represented in each row. (**B**–**E**) HSP70 (red) and APP (green) double staining in the dorsal cortex. While APP expression remains elevated through 7 d, HSP70 expression is comparatively decreased at 7 d. Few neurons are HSP70+/APP+. (**G**–**J**) ATF3 (red) and APP (green) double staining in the habenular nuclei. ATF3 follows the same expression pattern as HSP70, with decreased marker uptake at 7 d. In all cases, sham animals do not express any of the tested markers, with only the baseline marker uptake visible. Scale bar = 150 µm (Scale bar = 100 µm in image (**F**)).

**Figure 13:**
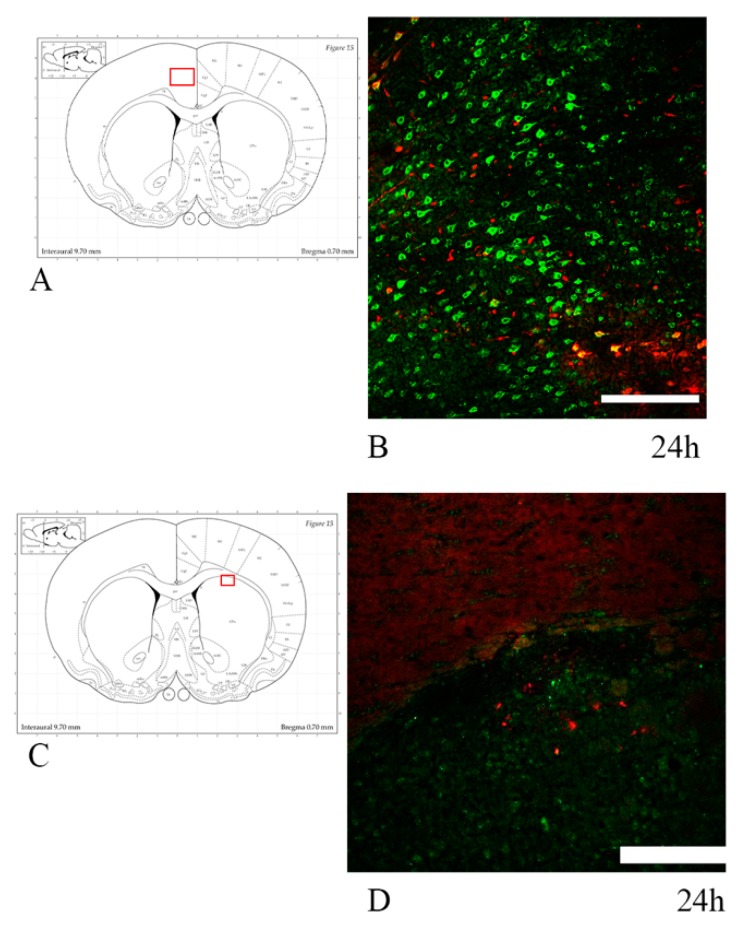
HSP70/APP double-staining in 24 h trauma-exposed animals. (**A**,**C**) The highlighted area refers to the area represented in the adjacent microphotograph. (**B**) HSP70 and APP expression in the cingulate cortex. Few neurons are positive for both markers. (**D**) HSP70 and APP expression in the caudoputamen, close to its interface with the corpus callosum. Scale bar = 200 µm.

HSP70 and ATF3 were expressed, in most cases, focally in the right dorsal cortex, both in frontal and in more caudal sections. An increased marker expression was also seen in the habenular nuclei and at the border of the hippocampal dentate gyrus with the stria medullaris (Figure 12). In one case minor HSP70 and ATF3 immunolabelling was noticed in the caudoputamen only. In one case, the cingulate cortex, somatosensory cortex and the corpus callosum showed an increased expression of the markers (Figure 13). The degree of neuronal stress—as indicated by HSP70 and ATF3 expression—seemed to be decreased by 7 d, with their expression being strongest at 24–72 h. Sham animals were negative for both HSP70 and ATF3. Only rare (ranging from zero to eight double positive neurons per 0.56 mm^2^) neuron cell bodies expressing HSP70 and ATF3 also showed abundant APP fluorescence. 

All the results—divided according to survival time and ROI—from investigations carried out in this study are summarized in Table 4.

## 4. Discussion

The objective of this study was to assess, for given survival times, how myelin and cells involved in its formation and degradation react to rotational brain injury. In particular, we assess the presence and location of axonal injury through the detection of APP expression, oligodendrocyte activity and distribution of their progenitor cells, through analysis of Olig2 (antibody and mRNA) and A2B5, NG2 antibodies, PDGFRa mRNA respectively. We also aim at assessing the post-injury myelination status by means of MBP and Rip expression and neuronal somata stress. This study continues the work by Davidsson et al. [46] on the understanding of DAI ensuing from sagittal angular acceleration trauma of the rat’s head. While qualitative analysis was performed through visual analysis of immunohistochemistry slides for all selected markers, quantitative analysis was performed by image analysis of IHC slides for APP and Rip expression, and of RNAscope^®^ slides for Olig2 and PDGFRa expression with Fiji ImageJ. RNAscope^®^ slides were chosen for quantitative analysis of Olig2 and PDGFRa for their clear expression of the investigated mRNA and ease of analysis through well-established protocols. These investigations were carried out by analyzing the specimen at 24 h, 72 h and 7 d after rotational TBI, in different brain areas (corpus callosum, dorsal cortex, habenular nuclei). The study was designed as such so as to insert the results (Table 4) within a specific timeframe and to estimate areas that are more susceptible to injury depending on their location in the frontal–caudal axis. The choice of such regions was based on their anatomical position with respect to the center of rotation, as a gradient of increasing injury severity can be observed moving further away from the skull base, in the frontal–caudal axis [5,49,50]. From a mechanical point of view, shear stress significantly affects areas that are found between tissues with different elasticity and white matter tracts that are oriented transversally to the plane of rotation [3,51,52]. This is of interest when trying to determine the threshold for axonal injury, myelin degradation and oligodendrocyte loss, i.e., for similar angular acceleration, different brain regions react differently, resulting in different degrees of injury.

### 4.1. Axonal Injury in TBI

Axonal injury is an important pathologic feature of TBI as it is associated clinically with unconsciousness, cognitive deficits, and generally poor outcome [7]. The pathophysiology of axonal injury is based on the mechanical damage to axons secondary to stretching and damage at a cellular level, primarily involving altered homeostasis and an increased calcium inflow resulting in axonal swelling and the accumulation of axonal transport proteins [4,53,54,55]. 

An APP antibody has been used as marker of axonal injury as it reflects the accumulation of the fast transport APP in the injured section of an axon, hence it is able to detect early axonal injury [12] and its pattern of expression (i.e., fusiform swellings, beaded and thick filaments), and distribution is specific to traumatic injury [56]. Differently from what the authors had done in previous studies investigating the same sagittal rotational acceleration model [46], APP immunostaining has been performed on formalin-fixed tissues because of the improved results shown during preliminary marker testing on both fresh and fixed tissues, especially when double stained with other markers (MBP, A2B5).

Quantitative and qualitative analysis of APP expression following rotational TBI confirmed what was expected from the results of previous studies. Throughout the 24 h–7 d survival time window, the analysis of APP expression in the frontal sections of the brain has shown APP-positive axon profiles localized mostly at the interfaces between white and gray matter tracts. This finding is consistent with the fact that the most susceptible areas to injury are those where there is a difference in tissue elasticity [3,5], allowing for the sliding of one region over the other during rotational insult, thus causing shear stress [4]. Instead, in the corpus callosum in the occipital units, APP expression revealed a decreased (*p* ≤ 0.05) amount of axonal injury (16.9 ± 7.10 APP+ profile) as compared to the frontal brain (37.6 ± 29.4 APP+ profiles).

This could be explained by the anatomical position: higher tissue shear stress and strain with higher risk of damage to the brain tissue is thought to occur more frontally than occipitally [5]. It might also be due to the fact that the occipital corpus callosum has a lower axon density [57]. The pattern of axonal injury produced by the rotational model of injury used in this study is in line with that produced by the rat finite-element model of rotation head trauma at similar levels of head acceleration [58]. Interestingly, Li et al. show opposite findings, i.e., APP is expressed abundantly primarily in the occipital corpus callosum, when investigating trauma produced by coronal rotation and lateral translation of the rats’ heads [59]. On the other hand, the degree of APP expression over time follows the same pattern as the one observed in this study: a decrease in APP expression by 7 d, after peaking at 24 h post-injury. This is a well-known pattern [59,60,61,62] and seems to occur due to increased concentrations of APP degrading enzymes within the injured axons, such as caspase-3 [63,64,65], as well as the recovery of mildly injured axons [66].

While the study included three older, heavier subjects (691.7 ± 56 g versus 409.4 ± 48.2 g), this did not significantly affect the degree of axonal injury resulting from the trauma, similarly to what was previously described for the same model of injury [67] (unpublished), and to eliminate potential biases due to weight differences, these three subjects were not included in the quantitative analysis, rather only in the preliminary qualitative analysis.

### 4.2. Oligodendrocyte Loss

Having confirmed that this rotational model of TBI induces diffuse axonal injury (DAI), we investigated the oligodendrocyte population to assess how they react to rotational TBI and whether they are robust enough to survive this type of relatively mild, diffuse injury. 

The presence of oligodendrocyte lineage cells in the regions of interest was studied through the anti-Olig2 antibody and mRNA expression. Olig2 is a transcription factor restricted to oligodendrocyte lineage and it is expressed early during development [35]. While Olig2 mRNA appeared to be less abundant and a trend of lower number of Olig2-expressing cells was noted in the trauma-exposed group as compared to the sham group, as well as from 24 h to 7 d survival times, Student *t*-test and one-way ANOVA test revealed these changes to be not statistically significant. This might be explained by the fact that the injury induced in this TBI model is not severe enough to cause oligodendrocyte loss or that the initial proliferation of OPCs is occurring, so that both non-mature and mature Olig2+ cells have been counted, offsetting the overall change in number of Olig2+ cells between TBI-exposed and sham animals [34]. In future studies, other techniques to better detect Olig2+ cell loss should be used, such as a different oligodendrocyte marker (e.g., CC1) and/or markers for apoptosis (e.g., caspase-3, TUNEL). Oligodendrocyte death following TBI is a known phenomenon, observed early following injury, between 12 h and 7 d for fluid percussion models [68,69], and in human studies [70]. Oligodendrocytes are vulnerable cells, sensitive to excitotoxicity (secondary to glutamate and other excitatory neurotransmitters release in DAI) and to oxidative stress (as, to sustain myelination process, they have high metabolic rates and consume high levels of ATP, but only have scarce concentration of glutathione), as well as to inflammatory cytokines, all conditions present in TBI [39,71].

### 4.3. Myelin Injury

The loss of oligodendrocytes leads to myelin damage and demyelination, leaving unmyelinated axons prone to injury and affecting the information processing speed [32,72], making the demyelination of intact axons possible [41]. Thus, enhancing oligodendrocyte survival and remyelination may offer ways of reducing post-TBI morbidity. 

Staining brain tissue with MBP antibodies has been widely used to study trauma-induced demyelination [21,73]; MBP expression is decreased when the myelin is damaged. 

This study indicated no decreased MBP expression as compared to baseline levels in sham. Such findings could fit with the hypothesis that myelin damage is more likely to be a hallmark of more severe TBI [25,33], as opposed to the milder TBI that is produced by the model used in this study. Moreover, the absence of MBP decreased expression and demyelination fits, with no significant oligodendrocyte loss found in the current study. Other authors report MBP expression is decreased following severe TBI in focal injury model: Liu et al. [21] claim that MBP is degraded ipsilaterally in the cortex and hippocampus between 2 and 72 h after controlled cortical impact (CCI). Ottens et al. [22] have also found, at 48 h after CCI injury, decreased expression of the same MBP isoforms as tested in this study. Yet, most authors have found evidence of demyelination starting at least 72 h, but more commonly up to 7 d, after CCI [25,41,74,75]. Wu et al.’s [60] finding of myelin sheath disruption—seen at electron microscopy—at 24 h in a model that combines angular and linear head acceleration. However, in the present study, no evidence of decrease in MBP expression is observed by 7 d.

Further investigations were conducted with anti-Rip as an alternative to MBP. Rip recognizes the CNP (2,3 cyclic nucleotide 3-phosphodiesterase) in mature, myelinating oligodendrocytes and stains the soma, cytoplasmic processes of oligodendrocytes and myelin sheaths—similarly to MBP [76,77]. Although by visual inspection Rip was expressed in a non-homogeneous fashion in the regions of interest both in sham and injured animals at any survival time, quantitative analysis (using mean grey value as parameter) revealed statistically significant (*p* ≤ 0.05) differences in Rip expression between sham and injured animals already at 24 h at the interface between CC and caudoputamen, and also at 7 d in the CC “SVZ”. This is in contrast to what other authors report, as decreased Rip expression was observed only by 7 d in a fluid percussion model of TBI [33]. Moreover, these results are confounding, as the mean grey value analysis shows higher values for the injured animals, while for demyelinating areas the authors expected to find decreased expression of Rip, which should have appeared darker, and thus mean grey values should have appeared lower. This hypothesis was supported by the results of other authors investigating Rip expression following TBI in areas such as fimbriae and external capsule [33]. A possible explanation for these results might be that when myelin is intact, Rip expression is strong and uniform, however, in a densely myelinated area such as the CC, this appears as background-like staining, which is reflected by low mean grey values. On the other hand, if—following injury—myelin is not compacted anymore, a decreased expression of Rip creates a visual contrast with its surrounding, so that the image appears brighter (Figure 11B) and it is translated into higher mean grey values. To test this hypothesis, mean grey value was calculated on the less densely myelinated caudoputamen, and this resulted in higher mean grey values than those found in the corpus callosum (MGV average in caudoputamen and CC of randomly chosen subjects of 15,283 and 13,653, respectively). These results seem to confirm that, in this study, areas of decreased Rip expression in injured animals—possibly corresponding to areas of myelin decompaction—are represented by higher mean grey values.

Nonetheless, further studies looking at myelin through electron microscopy would be needed to confidently assess the presence of demyelination in this model of injury. 

### 4.4. TBI-Induced OPCs Reaction 

The loss of mature oligodendrocytes and demyelination are major triggers for OPC activation and proliferation [36,78,79]. Immature glial precursor cells have been detected by staining with A2B5 marker [80,81]. An abundant presence of A2B5+ cells in sham groups and rotational TBI-exposed groups was observed in the subventricular zone (SVZ) at all investigated survival times. This finding may be explained by the fact that neural stem cells are maintained in the SVZ of the adult brain [82,83,84], while in basal conditions they lack the ability to give rise to glial cells during adulthood, myelin damage seems to be a stimulus for these glial precursor cells to proliferate, migrate in the injury area and differentiate in glial cells, contributing to myelin repair [85]. SVZ-derived progenitors (A2B5+ cells), together with parenchymal OPCs (NG2+ cells), are the main sources of oligodendrocyte progenitor cells in the adult brain [85,86]. The proliferation of SVZ-derived progenitor cells has been described as early as 48 h, with a peak at two weeks after direct demyelinating insult produced by focal TBI [87]. However, in this study we do not report evidence of A2B5+ cells proliferation up to 7 d. Similarly, Sullivan et al. [25] did not find the proliferation of SVZ progenitor cells up to 7 d post-TBI. In future studies, it might be worth investigating later timepoints to assess whether A2B5+ cell proliferation occurs—possibly in a delayed fashion—for the rotational TBI model used. For example, Chen et al. [61] found increasing numbers of SVZ precursor cells from six months after TBI, while Menn et al. [86] found a subset of these cells to be differentiated in mature oligodendrocytes and scattered through the corpus callosum and striatum at 21 days post-injury. Given the scarce amount of A2B5+ cells at all timepoints and regions of interest—except for at the SVZ—for all angular acceleration levels, attempts at correlating the presence of immature glial precursor cells with axonal injury have not produced any results.

Oligodendrocyte-restricted precursor cells were studied with an anti-NG2 antibody and PDGFRa mRNA. NG2 is a chondroitin sulfate expressed by OPCs [44,88]. However, it also stains pericytes, thus making it necessary to co-label it with Occludin, a tight-junction protein, to confidently distinguish the two NG2+ cells. While NG2 is commonly used as marker of OPCs, it is also expressed by glial cells differentiating into neurons or astrocytes, as well as macrophages [40,70,89,90,91,92]. For this reason, the analysis of NG2 immunostaining was only qualitative, supported by cell morphology observed at high magnification, while quantitative OPC analysis was based on PDGFRa mRNA. PDGFRa—platelet-derived growth factor receptor alpha—is a widely used marker for oligodendrocyte precursor cells in the central nervous system, as its expression—which occurs at about the same time as NG2—marks the OPC fate of the cell [70,93,94]. NG2+ OPCs were observed in all regions of interest and timepoints, at similar levels between TBI and sham animals, with no difference for increasing levels of angular head acceleration. Following oligodendrocyte death and demyelination, resident oligodendrocyte progenitor cells are activated and can proliferate, migrate and differentiate into mature oligodendrocytes, allowing for potential repair post-injury [37,39,95]. Thus, a thorough understanding of the mechanisms regulating oligodendrocyte death and OPCs activation is important to find therapeutic strategies to promote and improve recovery after TBI. Wide variability characterizes the phenomenon of OPC proliferation, as confirmed by the different results in different studies, using different models of TBI. Our rotational TBI study does not report and increased number of OPCs by 7 d. Instead, a marked increased number of NG2+ OPCs was found by other authors at the corpus callosum between 24 h and 7 d after CCI [25,96], while increasing number of OPCs were seen from 72 h to 7 d after fluid percussion TBI at the corpus callosum [25,40]. 

Considering that most authors report an increased number of NG2+ OPCs around 7 d post-TBI at the corpus callosum, the injury induced by our model of rotational TBI might not be severe enough to produce marked changes in OPC number. Moreover, the lack of OPC proliferation is to be expected in relation to the lack of oligodendrocyte loss and demyelination observed in the current study. In future studies, it might be of interest to look into the growth factors and cytokines (e.g., FGF2, TGFb) that regulate OPCs’ activation and proliferation [38], to know whether the cellular machinery is activated at all, and, if so, if evidence of an increased number of OPCs can be detected at later survival times. 

### 4.5. Neuronal Somata Stress

Diffuse axonal injury, as marked by APP expression, is thought to induce, at least transiently, neuronal cell body stress and damage [14]. Considering that our model of rotational TBI induces axonal injury mostly at the corpus callosum, in particular at the interface with the lateral ventricles and caudoputamen, we investigated the dorsal cortex for neuronal stress by means of HSP70 and ATF3, together with APP marker. HSP70 is part of the heat shock family, and it is strongly upregulated by a variety of stress stimuli (trauma, ischemia, heat, hypoxia), peaking at 24 h after exposure [16,18,19]. Similarly, ATF3 (activating transcription factor 3) is used as a cellular stress marker given its association with several pathways involved in cellular stress response [97,98]. Moreover, its induction has been well described in neurons, as well as in immune cells during neuroinflammatory processes, and occurs soon after the insult [99]. 

In six out of nine TBI-exposed animals (from the qualitative and quantitative analysis group), we found a focal area of injury superficially in the right dorsal cortex, in which neurons expressed HSP70, ATF3 and APP, in both frontal (about 1–1.3 mm bregma) and more caudal (about −3 mm bregma) brain sections. HSP70 and ATF3 expression was strongest at 24 h, but still moderate by 7 d. Other authors report expression of HSP70 and ATF3 up to 72 h following FP and CCI, and more extensively through the hippocampus, motor and somatosensory cortices [14,19]. The finding of focal injury is in contrast with the diffuse TBI typically produced by our model of injury, in which no such injury had been previously described [46,100]. Interestingly, Singleton et al. [14] did not find any HSP70 expression in the cortical area directly underlying the site of focal impact. The expression of HSP70/ATF3 and APP in deep structures such as the habenular nuclei and stria medullaris, the interface with the dentate gyrus of the hippocampus, indicates a possible multifactorial nature of neuronal cell body stress in the investigated rotational TBI model, which could be caused, to some extent, directly by axonal injury in the context of head angular acceleration, as well as neuroinflammation and axonal injury-mediated neuronal stress, and other secondary mechanisms, such as hypoxia [16,99].

## 5. Conclusions

This work gives an insight into white matter pathology following diffuse TBI induced by angular acceleration of the rat head. Diffuse APP expression—which was maximal at the white–gray matter border and greater in the frontal than the occipital regions throughout the 24–72 h time window and decreasing by 7 d—indicates that the rotational TBI model used in this study produces DAI for any angular acceleration between 0.96 and 2.2 Mrad/s^2^. While we detected moderate axonal injury, as indicated by stable expression of APP, and increased expression of neuronal stress markers, we found only minor trends of decreased oligodendrocyte lineage cell number and OPC proliferation, and mostly conserved myelination, as was visible through MBP and Rip immunostaining. This might indicate that the oligodendrocyte population could be robust enough to withstand the trauma ensuing from the delivered angular head acceleration. All in all, this confirms the mild severity of the investigated rotational TBI model. 

## Figures and Tables

**Table 1:** Information on the animals included in the study. The table summarizes information about the trauma, angular acceleration, survival time and weight of the animals included in the study. (**A**) refers to subjects who were included in both quantitative and qualitative analysis, while subjects in (**B**) were included only in preliminary qualitative assessments.

**A**			
**Subjects**	**Trauma Level**	**Survival Time**	**Weight ± SD (g)**
18 Sprague-Dawley rats	Sham → *n* = 9	24 h → *n* = 3	411.7 ± 17.9
		72 h → *n* = 3	429.7 ± 12.7
		7 d → *n* = 3	399.7 ± 18.6
	0.96–1.34 Mrad/s^2^ → *n* = 7	24 h → *n* = 3	444.7 ± 16.2
		72 h → *n* = 3	478 ± 88.7
		7 d → *n* = 1	376
	1.35–2.18 Mrad/s^2^ → *n* = 2	7 d → *n* = 2	393.5 ± 23.3
**B**			
**Subjects**	**Trauma Level**	**Survival Time**	**Weight ± SD (g)**
9 Sprague-Dawley rats	Sham → *n* = 2	24h → n = 1	372
		72h → n = 1	354
	0.96–1.34 Mrad/s^2^ → *n* = 1	72h → n = 1	749
	1.35–2.18 Mrad/s^2^ → *n* = 6	24h → n = 3	364.7 ± 9.0
		72h → n = 3	559.3 ± 181.4

**Table 2:** List of primary antibodies used in the study.

Antibody	Dilution	Supplier, Catalogue Number
Amyloid precursor protein (APP)	1:200	Invitrogen, 51-2700
Myelin Basic Protein (MBP)	1:200	Merk Millipore, Ne1019
Rip	1:300	DSHB
Olig2	1:500	AbCam, Ab109186
NG2	1:200	Millipore, A25320
Occludin	1:100	Santa Cruz, Sc-8145
A2B5 (4D4 A2B2-like-supernatant)	1:50	DSHB
HSP70	1:200	Santa Cruz, Sc-66048
ATF3	1:100	Santa Cruz, Sc-81189

**Table 3:** List of RNAscope^®^ probes.

Target mRNA Probe	Nucleotides	Accession Number
Olig2	375-1986	NM_001100557.1
PDGFRa	1516-2531	NM_012802.1

**Table 4:** Summary of results. Changes in Olig2 and PDGFRa are not statistically significant. + refers to expression of the antibody at similar levels as the sham; - refers to no expression of the antibody, similarly to sham; ↑ refers to increased expression of the antibody, as compared to sham; ↓ refers to decreased expression of the antibody, as compared to sham; N.I. means that the ROI was not investigated for that specific antibody. * changes observed in only one subject.

Survival Time	ROI	APP	MBP	Rip	OLIG2	A2B5	NG2	PDGFRa	HSP70	ATF3
24 h	CC “SVZ”	↑	+	↑	+	+	+	+	-	-
	CC “BORDER”	↑	+	↑	+	-	+	+	↑ *	-
	CC “OCCIPITAL”	↑	+	+	↓	-	+	↑	↑ *	-
	DORSAL CORTEX	↑	N.I.	N.I.	N.I.	N.I.	N.I.	N.I.	↑	↑
	HABENULAR NUCLEI	↑	N.I.	N.I.	N.I.	N.I.	N.I.	N.I.	↑	↑
72 h	CC “SVZ”	↑	+	+	+	+	+	+	-	-
	CC “BORDER”	↑	+	+	↓	-	+	↓	-	-
	CC “OCCIPITAL”	↑	+	+	↓		+	↑	-	-
	DORSAL CORTEX	↑	N.I.	N.I.	N.I.	N.I.	N.I.	N.I.	↑	↑
	HABENULAR NUCLEI	↑	N.I.	N.I.	N.I.	N.I.	N.I.	N.I.	↑	↑
7 d	CC “SVZ”	↑	+	↑	+	+	+	↑	-	-
	CC “BORDER”	↑	+	+	+	-	+	↓	-	-
	CC “OCCIPITAL”	↑	+	+	↓	-	+	↑	-	-
	DORSAL CORTEX	↑	N.I.	N.I.	N.I.	N.I.	N.I.	N.I.	↑	↑
	HABENULAR NUCLEI	↑	N.I.	N.I.	N.I.	N.I.	N.I.	N.I.	↑	↑
